# Modulation of Nerve Cell Differentiation: Role of Polyphenols and of Contactin Family Components

**DOI:** 10.3389/fcell.2019.00119

**Published:** 2019-07-18

**Authors:** Sabrina Picocci, Antonella Bizzoca, Patrizia Corsi, Thea Magrone, Emilio Jirillo, Gianfranco Gennarini

**Affiliations:** ^1^Laboratories of Developmental Neurobiology, Department of Basic Medical Sciences, Neurosciences and Sensory Organs, Medical School, University of Bari Aldo Moro, Bari, Italy; ^2^Laboratories of Immunology, Department of Basic Medical Sciences, Neurosciences and Sensory Organs, Medical School, University of Bari Aldo Moro, Bari, Italy

**Keywords:** contactins, gene regulation, neural development, Notch pathway, polyphenols

## Abstract

In this study the mechanisms are explored, which modulate expression and function of cell surface adhesive glycoproteins of the Immunoglobulin Supergene Family (IgSF), and in particular of its Contactin subset, during neuronal precursor developmental events. In this context, a specific topic concerns the significance of the expression profile of such molecules and their ability to modulate signaling pathways activated through nutraceuticals, in particular polyphenols, administration. Both *in vitro* and *in vivo* approaches are chosen. As for the former, by using as a model the human SH-SY5Y neuroblastoma line, the effects of grape seed polyphenols are evaluated on proliferation and commitment/differentiation events along the neuronal lineage. In SH-SY5Y cell cultures, polyphenols were found to counteract precursor proliferation while promoting their differentiation, as deduced by studying their developmental parameters through the expression of cell cycle and neuronal commitment/differentiation markers as well as by measuring neurite growth. In such cultures, Cyclin E expression and BrdU incorporation were downregulated, indicating reduced precursor proliferation while increased neuronal differentiation was inferred from upregulation of cell cycle exit (p27^–Kip^) and neuronal commitment (NeuN) markers as well as by measuring neurite length through morphometric analysis. The polyphenol effects on developmental parameters were also explored *in vivo*, in cerebellar cortex, by using as a model the TAG/F3 transgenic line, which undergoes delayed neural development as a consequence of Contactin1 adhesive glycoprotein upregulation and premature expression under control of the Contactin2 gene (*Cntn-2*) promoter. In this transgenic line, a Notch pathway activation is known to occur and polyphenol treatment was found to counteract such an effect, demonstrated through downregulation of the Hes-1 transcription factor. Polyphenols also downregulated the expression of adhesive glycoproteins of the Contactin family themselves, demonstrated for both Contactin1 and Contactin2, indicating the involvement of changes in the expression of the underlying genes in the observed phenotype. These data support the hypothesis that the complex control exerted by polyphenols on neural development involves modulation of expression and function of the genes encoding cell adhesion molecules of the Contactin family and of the associated signaling pathways, indicating potential mechanisms whereby such compounds may control neurogenesis.

## Introduction

Adhesive glycoproteins modulate different events of neurogenesis, including precursor proliferation, commitment, and differentiation (Missaire and Hindges, 2015). In the context of this regulatory role a special concern is on those pathways, which mediate the developmental control of such events (Pollerberg et al., 2013; Frei and Stoeckli, 2014; Stoeckli, 2018). Such pathways may imply the function of cell surface adhesive glycoproteins belonging to different gene families as for instance the Cadherins and the Semaphorins (Hirano and Takeichi, 2012; Jongbloets and Pasterkamp, 2014) and, in this study, we focus on components of the large Immunoglobulin Supergene Family (IgSF; Dityatev et al., 2008; Stoeckli et al., 2013; Frei and Stoeckli, 2017) and, mostly, on its Contactin subset with a special focus on Contactin1 (Stoeckli, 2010, 2018; Xenaki et al., 2011; Zuko et al., 2011, 2013; Puzzo et al., 2013, 2015; Mohebiany et al., 2014; Gennarini and Furley, 2017; Gennarini et al., 2017).

On a mechanistic ground, the Contactin1 function implies its ability to interact with and to modulate developmental control pathways, among which a pivotal role is played by the one associated with Notch receptors (Louvi and Artavanis-Tsakonas, 2006; Zhang et al., 2018). Indeed, besides by the Delta and Serrate canonical ligands (Wang, 2011), Notch receptors activation may also result upon their interactions with non-canonical ligands and a relevant role in this respect has been demonstrated for components of the Contactin family (Hu et al., 2003, 2006; Bizzoca et al., 2012; Gennarini et al., 2017). Contactins are glycosylphosphatidylinositol (GPI)-anchored cell surface glycoproteins, which include six components, labeled from Contactin1 to Contactin6. These molecules share a similar overall organization as they are built by structurally related domains, which may belong to either the Immunoglobulin type C2 and/or the Fibronectin type III subtypes, whose variable association generates the IgC2/FNIII IgSF subset (Ranscht, 1988; Gennarini et al., 1989, 1991; Shimoda and Watanabe, 2009). While bearing comparable overall organization and functional roles at the cellular level, a specific developmental significance for such molecules relies upon the differential activation profiles of the underlying genes, which, in turn, may sustain their complex interactions via either homo- or heterophylic mechanisms (Mohebiany et al., 2014). In these molecules, the association of immunoglobulin type C2 with Fibronectin type III domains is justified by their similar organization (Cota et al., 2001), which, in turn, is relevant for those mechanisms, which are involved in developing nervous tissue patterning. Indeed, IgC2/FNIII proteins expression profiles and molecular interactions are both ontogenetically regulated, which may be relevant for their ability to activate developmental control pathways (Gennarini et al., 2017).

Close relationships in terms of structural organization, expression profiles and functional roles have been originally demonstrated in the case of the first two identified members of the IgC2/FNIII subset, the Contactin1 and the Contactin2 glycoproteins, thanks to the use of *in vitro* models (Ranscht, 1988; Gennarini et al., 1989; Furley et al., 1990). These studies indicated that differential activation of the underlying genes during either early or, respectively, late ontogenesis may contribute to their specific ontogenetic function. Accordingly, changes in the profiles of such genes may result in morphogenetic consequences in regions provided with different developmental history, including the cerebellar and cerebral cortices (Bizzoca et al., 2003, 2012, 2015), the basal ganglia (Massaro et al., 2012), and the hippocampus (Puzzo et al., 2013, 2015). In addition, these changes may sustain the pathogenesis of specific neurological disorders, typically shown in the case of the autism (Zuko et al., 2011, 2013) and of some dysmyelinating neuropathies (Querol et al., 2013; Manso et al., 2016).

The above evidences support the relevant significance of the mechanisms, which drive regulated expression of Contactin axonal morphoregulatory molecules in nervous tissue differentiation and patterning. A remarkable example in this respect is provided by the molecular interactions of the Contactin1 adhesive glycoprotein, which, besides further adhesion molecules (Gennarini and Furley, 2017; Gennarini et al., 2017), may also involve the products of developmental control genes. Among them a special functional and developmental significance has been assigned to the Contactin1 interactions with Notch receptors (Hu et al., 2003; Bizzoca et al., 2012; Gennarini et al., 2017; Zhang et al., 2018), which, during nervous tissue patterning, may be mostly relevant for modulating key developmental events as axonal growth and pathfinding, typically shown in cerebellar (Bizzoca et al., 2003) and cerebral (Bizzoca et al., 2012) cortices and in the hippocampus (Gulisano et al., 2017), the latter also involving modulation of the expression of the pCREB transcription factor (Puzzo et al., 2013, 2015). Therefore, via differential molecular interactions (with Notch and/or pCREB), the same morphoregulatory molecule may exert a complex and articulated control on either early or, respectively, late neural development, thus strengthening the relevance of the mechanisms, which drive the regulated expression of the underlying genes.

The present study will focus on such molecular interactions, but also on the associated signaling pathways, which, again, may be activated during both early and late developmental stages. In addition, a special emphasis will be on the significance of components of the dietary intake, and in particular, on polyphenols, given their ability to modulate neurogenesis (Valenti et al., 2016; Ding et al., 2017; Poulose et al., 2017; Bensalem et al., 2018; Sarubbo et al., 2018). Indeed, in the nervous tissue, relevant polyphenol effects are demonstrated during development, but also during aging (Bensalem et al., 2018) and in those conditions characterized by tissue damage (Renno et al., 2017), consistent with their demonstrated ability to support phenotype recovery in neurodegenerative/neurodevelopmental disorders (Syarifah-Noratiqah et al., 2018). On a mechanistic ground, these effects may be related to the polyphenols antioxidant activity (Lalkovièová and Danielisová, 2016), thus supporting the ability of such compounds and of the downstream pathways to exert neurotrophic effects (Moosavi et al., 2015). In this respect, a typical example is provided by grape seed polyphenols (GSP), whose ability to promote neural regeneration has been demonstrated in different studies (He et al., 2017; Renno et al., 2017; Ruzicka et al., 2018), as specifically shown in the case of the developmental events of cochlear hair cells (Gu et al., 2015). The polyphenol antioxidant activity may therefore represent a relevant and appropriate therapeutic tool toward the mentioned conditions (Abolhassani et al., 2017; Ahmed et al., 2017; Aguilera et al., 2018).

Polyphenols are ubiquitously distributed, as they are present in fruits, vegetables, cereals, and extra virgin olive oil (Magrone and Jirillo, 2018). Chemically, they may be divided into two major classes, flavonoids and non-flavonoids (e.g., resveratrol), which may exert their biological functions in different tissues and organs, including the nervous and the immune systems, in which they control the peripheral immune response on both the innate or the adaptive arms of the immune machinery (Magrone et al., 2007, 2008a). In the immune system these compounds are known to counteract the NF-κB pathway and as such they may induce a tolerogenic anti-inflammatory pathway as demonstrated either *in vitro* or *in vivo* (Magrone et al., 2008b, 2017a; Marzulli et al., 2014). Within the central nervous system (CNS), polyphenol anti-oxidant and anti-inflammatory effects should be taken into account, according to their mentioned capacity to promote an immune tolerogenic pathway (Magrone et al., 2012, 2017b). In Parkinson’s disease (PD) neuronal damage mediated by 5-S-cysteinyldopamine is markedly attenuated by quercetin, hesperetin and caffeic acid, which are derivatives of catechin and epigallocatechin (Mandel and Youdim, 2004; Spencer, 2009). Moreover, quercetin anti-inflammatory effects on glial cells also prevent neuronal death (Hunot and Hirsch, 2003; Spencer, 2007). The polyphenol protective effects against neurodegeneration are consistent with their therapeutic role on specific neurological disorders as is the case for the Alzheimer’s disease (AD) in which they afford neuroprotection *via* activation of brain-derived neurotrophic factor (BDNF). In AD, resveratrol promotes neuroprotection *via* breakdown of the amyloid precursor proteins and removal of neurotoxic Aβ peptides (Deng and Mi, 2016). In addition, polyphenols may act through the Sirt pathway, whose reduced levels in AD were found to enhance NF-kB expression, thus counteracting inflammation and Aβ toxicity (Almeida et al., 2016; Gao et al., 2016). A neuroprotective mechanism may also rely upon the polyphenol ability to counteract expression of granzymes in cytotoxic T cells (Tc), thus preventing neuronal damage by Tcs infiltrating the CNS (Magrone et al., 2012). Similarly, polyphenols may also induce activation of FoxP3^+^ CD4^+^ T cells, the so called T regulatory (Treg) cells, which are producers of the anti-inflammatory (IL)-10 interleukin cytokine (Marzulli et al., 2012; Magrone et al., 2017a, c). In turn, Treg cells migrating from periphery to CNS may dampen neuroinflammation in the course of PD and AD, respectively.

Taken together, the above data indicate that polyphenols share in common with IgC2/FNIII molecules the ability to counteract neurodevelopmental delay and neurodegeneration and, based on these considerations, a relevant aim of the present study is to validate the potential significance of IgC2/FNIII molecules cooperation with polyphenols in exerting protective effects against neuronal damage. For this, in the present study transgenic mice undergoing IgC2/FNIII molecules misexpression are used as animal models, in which the potential polyphenol protective effects against neurodegeneration are explored. Both *in vivo* and *in vitro* approaches will be used in this attempt. As for the former, mutant mice undergoing changes in the regulated expression profile of such molecules have been chosen. As for the *in vitro* models rather than primary neural cultures, cell lines of neural derivation have been selected, with reference to the SH-SY5Y neuroblastoma cells, the reason for this being that this cell line has been extensively used in studies focusing on neuronal precursor proliferation and differentiation (see Xie et al., 2010; Singh et al., 2017) and, in addition, that using this line is much easier than using primary cultures, while providing comparable information. Moreover, concerning the specific topic of this study, this line was found to undergo differentiation upon polyphenol treatment (Kalfon et al., 2007) and therefore it represents a useful, quick and inexpensive model for exploring developmental parameters at the cellular level upon polyphenol stimulation.

## Materials and Methods

In the present study both *in vitro* and *in vivo* models have been used.

### *In vitro* Models

The SH-SY5Y neuroblastoma line has been chosen to evaluate the potential effects of polyphenol administration on the neuronal phenotype. This cell line was purchased from ATCC (Manassas, VA, United States) and cultivated at 37°C in Dulbecco’s Modified Eagles Medium (DMEM), supplemented with 10% v/v fetal bovine serum (FBS), containing 1% penicillin/streptomycin and 2 mM L-glutamine in a 5% CO_2_ atmosphere. For the attempts of the present study the cells were seeded at an initial density of 10^4^/cm^2^, and cultivated for 24 h before starting polyphenols treatment, which consisted in red grape extract (GSP) addition to the cultures (see below).

#### Polyphenol Treatment

As for the polyphenol source, dry extracts containing both proanthocyanidins and catechins were courtesy provided by Farmalabor s.r.l. (Canosa di Puglia, Italy). GSP were extracted from *Vitis vinifera* grape cultivar (Apulia, South Italy) by percolation with ethanol:water (70:30). They were added to SH-SY5Y cell cultures to final concentrations from 1 to 100 μg/ml in the medium, starting from a 1 mg/ml stock in DMSO. The lowest concentration affecting the growth rate was established by building a dose-dependent curve in which the GSP effects on SH-SY5Y cells proliferation were measured by the MTT assay (Giordano et al., 2011) over 3 days treatments.

#### Cell Growth Rate Estimation

SH-SY5Y cells were originally maintained as frozen stocks or as stocks of living cells. To estimate the effects of GSP administration on their proliferation/differentiation parameters they were seeded on 18 mm coverslips in six wells plates and GSP was then added to the medium 24 h later. At different time points (1, 2, 3, 5, 7, and 12 days) the number of viable cells was estimated through a trypan blue exclusion test and expressed as the percentage of the value observed in controls.

#### Time Course of Cell Cycle Progression

To follow the time course of cell cycle progression over the indicated time lapse, cell proliferation was evaluated through different cell cycle markers. GSP was added to the medium 24 h after plating at a 1 μg/ml while BrdU was added at a 20 μM concentration. Cells were then incubated for 2 h, washed, fixed by 4% paraformaldehyde (PFA) and finally saturated in PBS/2% milk for 1 h at RT. The coverslips were then incubated with BrdU antibodies (mouse monoclonal, Roche Molecular Biochemicals, Mannheim, Germany), overnight at 4°C and with primary antibodies against p27^kip1^ (mouse monoclonal, BD Biosciences, San Jose, CA), Cyclin E (rabbit polyclonal, Abcam, Cambrige, MA, United States), β-tubulin (mouse monoclonal, Novus Biologicals, Centennial, CO, United States), followed by secondary antibodies coupled to Alexa Fluor 488 or 568 (Jackson ImmunoResearch Laboratories Inc., West Grove, PA, United States). Nuclei were counterstained with DAPI and, after immunostaining, cells were examined by a TCS SP8 confocal laser-scanning microscope (Leica Microsystems, Wetzlar, Germany) through a sequential procedure. Confocal images were taken at 1 μm intervals through the *Z*-axis of the sections at a 20× magnification and Z-stacks of serial optical planes were then analyzed by a Leica confocal software (Multicolour Package, Leica Microsystems).

Labeled cells were automatically counted by using the ImageJ software (Schneider et al., 2012) and their density expressed as their percentage toward the overall cell number, estimated by DAPI staining. The corresponding images were digitized, optimized by contrast enhancement functions and segmented by an interactive modality of the program. For the different markers, the program also allowed to select the same threshold for each group. Quantitative estimation of neuronal precursor commitment/differentiation, as well as histomorphometric measurements were similarly done on immunostained slides. The resulting binary images were then processed by functions of the Neurite Tracer plug-in program allowing measurement of neurite growth (Pool et al., 2008).

### *In vivo* Models

Developmental parameters were also studied *in vivo* in the cerebellar cortex, in which the GSP effects were explored in wild type (WT) or, alternatively, in TAG/F3 transgenic mice, undergoing Contactin1 developmental overexpression under control of the Contactin2 promoter (Bizzoca et al., 2003). The generation and use of this line have been described (Bizzoca et al., 2003, 2012, 2015; Coluccia et al., 2004; Puzzo et al., 2013, 2015). TAG/F3 mice were maintained as heterozygotes and time-pregnant littermates were used throughout, the day of vaginal plug being considered as the embryonic day 0 (E0). For polyphenols, a mice diet consisting in food mixed with grape seed extracts (92.5 mg of polyphenols/gr of food), was administered *ad libitum*. Mice fed in the absence of grape extract were used as the controls. The treatment begun during the embryonic life by GSP administration to the pregnant mothers; polyphenol-containing or control diets were then maintained until the end of the first postnatal week when the animals were sacrificed for examination. At each developmental stage, the study included four groups of mice: WT and transgenic (TAG/F3), fed with either conventional or supplemented food.

#### Immunostaining

For immunohistochemical procedures, mice perfused with 4% PFA in 0.12M phosphate buffer (PB) pH 7.4 were dissected and the tissues were post-fixed overnight in the same fixative. Twelve micrometers cryostat sections were then generated and immunostainings were done on sagittal sections from the cerebellar vermis, whose derivation along the medio-lateral axis was deduced upon comparison with adjacent structures. For the *in vitro* data, cultures from the SH-SY5Y human neuroblastoma line were similarly fixed and immunostained as above.

For Contactin1 immunostaining, the LIM antiserum, raised in rabbits against the 20 N-terminal amino acid sequence of the molecule and reproducing its expression profile (Virgintino et al., 1999; Bizzoca et al., 2003, 2009, 2012; De Benedictis et al., 2006) or, alternatively, a mouse monoclonal Contactin1 antibody (Chemicon, Temecula, CA) were used. Contactin2 was detected through a rabbit antiserum (Dodd et al., 1988) and NeuN through a mouse monoclonal antibody from Chemicon. As for Hes-1, a rabbit antiserum was obtained from Abcam.

To estimate precursor proliferation *in vivo*, mice were intraperitoneally injected with 5-bromo-2′-deoxyuridine (BrdU, Roche Molecular Biochemicals, Mannheim, Germany, 50 ug/g body weight) and sacrificed 5 days later when BrdU was detected through a sheep antiserum from Novus Biologicals.

For immunostaining, the same section thickness, antibody concentrations, incubation time, developing conditions, microscopic pictures acquisition and handling procedures were applied at all developmental stages. Sections permeabilized for 30′ at RT with 0.5% Triton X-100, 3% BSA, 5–10% FCS were incubated ON at 4°C with primary antibodies diluted in PBS containing 3% BSA, 5–10% FCS and 0.25% Triton X-100, followed by secondary antibodies (1 h incubation at RT).

Immunostained sections were analyzed by using a TCS SP8 confocal laser-scanning microscope (Leica) through a sequential scanning procedure. Confocal images were taken at 1 μm intervals through the *z*-axis of the sections with 20× or 40× lenses. Z-stacks of serial optical planes were analyzed by a Leica confocal software (Multicolour Package, Leica Microsystems). BrdU- and NeuN-labeled cells were automatically counted by the ImageJ software, and their density expressed as their percentage over the overall cell number, estimated by DAPI staining. Histomorphometric measurements for Hes-1, Contactin1, Contactin2, and NeuN were done on selected corresponding sections by the ImageJ software. Corresponding images were digitized, optimized by contrast enhancement functions and segmented by an interactive modality of the program. For each marker, the program also allowed to select the same threshold for each group. The resulting binary images were then processed by functions allowing measurement of the extent of the immunostaining, deduced by the pixel number.

#### Animal Breeding

Mice were bred in the Department of Basic Medical Sciences, Neurosciences and Sensory Organs, University of Bari Aldo Moro, Italy and experimentation conformed the EU directive 2010/63/EU by following the Italian Ministry of Health law of March 4, 2014, n. 26 upon the Authorization n. 982/2016 released from the Italian Ministry of Health on October 17, 2016, which also included the ethical approval of the proposed research from the “Organism in charge of animal welfare” of the Bari University.

#### Statistical Analyses

All experiments were performed in triplicate and repeated at least three times. In order to identify statistically relevant data, the differences were evaluated by ANOVA tests or by the Student’s *t* test. The mean values ± standard error mean (SEM) were calculated to represent the three experiments. A *p*-value < 0.05 was considered statistically significant. As for the distribution of the obtained data, this was explored by using the GraphPad program, which also allowed *P* values correction.

As for the *in vitro* experiments aiming at following cell cycle progression (BrdU incorporation, Cyclin E, p27 Kip expression) or neuronal differentiation (β-tubulin expression), the data were expressed as the mean and the SEM. Such data approached a Gaussian distribution and respected assumptions for parametric tests, so that comparisons between independent groups were performed by *t*-tests. Each measure was repeated at the different days and adjustments for multiple testing were applied according to Bonferroni. In experiments implying dose-dependent effects a one-way ANOVA test was used, while two-way ANOVA was chosen for cell growth rate measures. The following variables: “cerebellar size,” “NeuN,” “Contactin2,” and “BRDU” approached a Gaussian distribution and a linear model; therefore a two way factorial ANOVA model with interaction was applied to evaluate the effects of treatment (treated vs. control) and gene expression (WT vs. TAG/F3) and their interaction. Results were described as least square means and their 95% confidence intervals. *P*-values for *post hoc* comparisons were adjusted according to Tukey.

Contactin1 expression did not approach a Gaussian distribution and diverge from a linear model, therefore data were summarized as median and 95% confidence interval of the median. Since, we had two factors (gene expression and polyphenol treatment) with two levels, as previously described for other variables, we needed to perfom, by Mann–Whitney *U* test, eight pairs comparisons of indipendent groups. Given the multiple testing procedure, to conclude about effects of treatment and of transgene expression *p*-values were adjusted according to Bonferroni.

## Results

### *In vitro* and *in vivo* Models for Studying the Neurodevelopmental Consequences of Contactin1 Overexpression and of Polyphenol Administration

This study includes two distinct, although overlapping sections, dealing, respectively with *in vitro* and *in vivo* approaches for exploring the significance of adhesive glycoproteins expression profile and of polyphenol administration in the control of nervous tissue developmental events. First the *in vitro* results obtained in the SH-SY5Y neuronal cell line will be reported. Then the *in vivo* data arising in transgenic mice undergoing Contactin1 developmental overexpression driven from the regulatory region of the gene encoding the Contactin2 glycoprotein (also called Transient Axonal Glycoprotein TAG-1, Furley et al., 1990), the TAG/F3 transgenic line (Bizzoca et al., 2003), will be discussed.

In the SH-SY5Y neuronal cell line (Cuevas et al., 2015; Saeed et al., 2015; Zheng et al., 2015) the effects of GSP on proliferation/commitment/differentiation parameters, as well as on cell death, were originally estimated. SH-SY5Y cells have been extensively used in studies aiming at evaluating neuronal precursor proliferation and differentiation parameters (see Xie et al., 2010). Moreover, as far as the specific topic of this study is concerned, previous studies demonstrated that this cell line undergoes differentiation upon polyphenol treatment (Kalfon et al., 2007) and as such it represents an useful tool for exploring developmental parameters at the cellular level. Finally, a relevant advantage of focusing on this line rather than on primary neurons is on its ability to undergo large-scale expansion upon relatively easy culture conditions (Xicoy et al., 2017). For such an *in vitro* approach, cells bearing the N-type morphology were used, which allowed exploring their phenotype in terms of developmental events, mostly related to neuronal differentiation.

As indicated the *in vivo* developmental significance of the Contactin adhesion molecules expression profile was explored by using the TAG/F3 transgenic line (Bizzoca et al., 2003, 2012), which undergoes Contactin1 overexpression and premature expression under control of the Contactin2 gene (*CNTN-2*) promoter. Previous studies revealed that expressing the TAG/F3 transgene in developing nervous tissue resulted into an ontogenetic delay, mostly evident in the cerebellum (Bizzoca et al., 2003), in the cerebral cortex (Bizzoca et al., 2012), in the basal ganglia (Massaro et al., 2012) and in the early developing hippocampus (Puzzo et al., 2013, 2015). As for the underlying mechanisms, these effects could be attributed to the Contactin1 ability to activate the Notch pathway (Hu et al., 2003, 2006; Bizzoca et al., 2012), known to result into a neurodevelopmental delay (Zhang et al., 2018). In the same context, Contactin1-overexpressing mice provided a suitable model to explore the ability of polyphenol administration in counteracting neurodevelopmental delay. In addition, this transgenic line was especially useful for exploring the molecular mechanisms underlying neuronal differentiation, with reference in particular to genetic (adhesion molecules expression) and epigenetic (polyphenol administration) factors. Therefore, in the proposed experimental models, the significance of different factors, known to modulate neural developmental events was evaluated by the use of either *in vitro* and *in vivo* approaches as reported below.

#### *In vitro* Approach: The SH-SY5Y Cell Line

The transformed neuron-like cell line of human derivation SH-SY5Y (ATCC-CRL-2266) originates from a metastatic human bone tumor biopsy and represents a subline of the parental SK-N-SH cells, often used in studies focusing on neurodegenerative disorders and aiming at devising therapeutic strategies (Singh et al., 2017). In the context of the present study, the interest of this poorly differentiated line was also based on the evidence that it can be driven into a more mature phenotype upon polyphenol treatment (Kalfon et al., 2007). Therefore, a preliminary characterization of the proliferation/differentiation parameters of the SH-SY5Y line upon polyphenol treatment was done.

##### Effects of GSP treatment on SH-SY5Y cell phenotype

**FIGURE 1:**
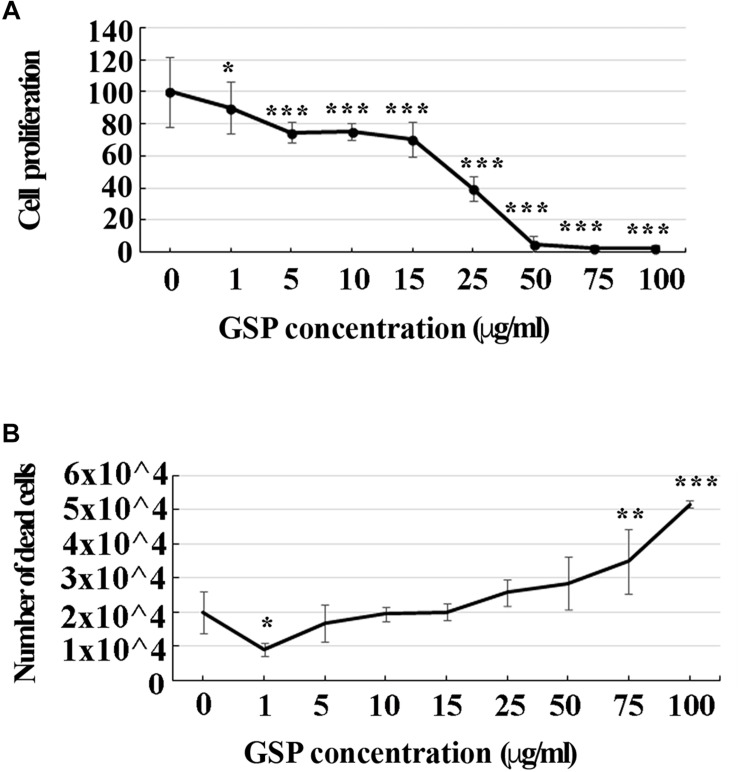
GSP treatment affects SH-SY5Y cells proliferation and survival. **(A)** SH-SY5Y cell proliferation: treatment for 72 h induced a GSP concentration-dependent reduction of SH-SY5Y cell proliferation, deduced by the cell density and measured through the MTT assay. For statistical analysis a one-way ANOVA test was used: ^*^p < 0.05; ^∗∗∗^*p* < 0.0001. Values are expressed as mean ± SEM. **(B)** SH-SY5Y cell survival: GSP treatment affected cell viability by inducing a dose-dependent increase in the number of dead cells. Low doses (1–5 μg/ml) led to only minor effects while high doses (75–100 μg/ml) were toxic, resulting in dose-dependent cell death. Dead cells were detected by Trypan blue exclusion tests. Values represent averages of triplicate measurements. As in panel **(A)**, statistical analysis was performed by using one-way ANOVA test: ^*^p < 0.05; ^∗∗^*p* < 0.001; ^∗∗∗^*p* < 0.0001.

To look at the best working conditions for polyphenol treatment, a dose-dependent curve of SH-SY5Y cell proliferation was first established by estimating their growth profile upon increasing GSP concentration in the medium (1–100 μg/ml upon 3 days treatment steps). As shown in Figure 1A, increasing the GSP input resulted into a reduced SH-SY5Y cell proliferation, expressed as the percentage of control cells, which was virtually abolished when GSP concentrations exceeding 50 μg/ml were reached, these data indicating inhibitory GSP effects on SH-SY5Y cell proliferation.

To verify whether apoptosis contributed to these effects, the number of dead cells was also estimated through a Trypan Blue exclusion test upon an increasing GSP input. As shown in Figure 1B, increasing polyphenol concentrations from 1 to 100 μg/ml significantly expanded the number of dead cells, indicating that, besides exerting anti-proliferative activity, GSP also affected cell survival, thus leading to cell loss. For the statistical evaluation of all the above data a one-way ANOVA test was used.

##### Cell growth rate

**FIGURE 2:**
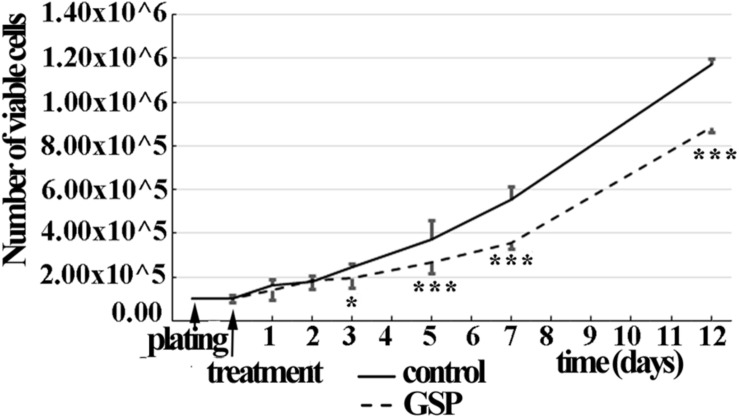
GSP treatment counteracts SH-SY5Y neuroblastoma cell growth. Cell growth was determined by estimating the number of viable cells by a Trypan Blue exclusion test at days 1, 2, 3, 5, 7, and 12 after plating. At each developmental stage, the data are representative of at least three experiments. Values are expressed as the mean ± SEM. Statistical analysis was done through a two-way ANOVA test: ^*^p < 0.05; ^∗∗∗^*p* < 0.0001.

The time course of SH-SY5Y cell proliferation was also deduced through the overall number of viable cells in a wider time lapse (1–12 days) in the presence/absence of GSP in the medium (1 μg/ml concentration). As shown in Figure 2, starting at the third day of treatment, a significant decrease in viable cells was observed in GSP-treated versus control samples by a trypan-blue exclusion test, thus supporting that an effect on cell viability contributed to the observed phenotype. For these data two way ANOVA test and Dunnet’s multiple comparison test were used.

##### Cell cycle progression

**FIGURE 3:**
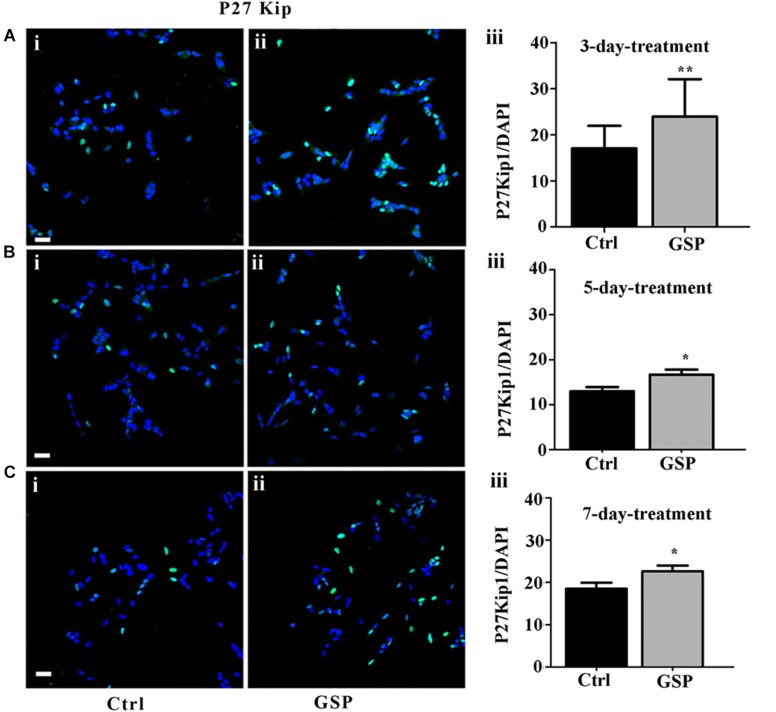
GSP treatment promotes SH-SY5Y neuroblastoma cell cycle exit. Cell cycle exit of SH-SY5Y cells was measured through the expression of the cyclin-dependent kinase inhibitor p27^Kip1^. In panels **(A–C)**, double stainings for p27^Kip1^ and DAPI are reported in GSP-treated **(ii)** versus control **(i)** cells upon 3 **(A)**, 5 **(B)**, and 7 **(C)** days in culture. The bar graphs shown in panels **(A–C,iii)** report the p27^Kip1^/DAPI ratio in both conditions. The data are representative of at least three experiments and are expressed as the mean ± SEM. Statistical analysis was done through a *t*-test: ^*^p < 0.05; ^∗∗^*p* < 0.001. Scale bars: 20 μm.

To confirm the contribution of changes in precursor proliferation to the overall cell number, cell cycle progression was also explored through cell cycle markers expression, known to be modulated by polyphenol administration and to result in specific effects on cell cycle exit at the G1 phase, in particular at the level of G2/M interphase transition (Shin et al., 2013). To explore these parameters the GSP effects on cell cycle were evaluated through the expression of p27^kip1^, a kinase inhibitory protein (KIP) family member known to drive precursors toward G0 at the G1/S interphase (Polyak et al., 1994; Hnit et al., 2015). Based on the reported time course for cell growth (Figure 2), 3, 5, and 7 days cultures were analyzed. At these time points p27^Kip1^ levels were morphometrically estimated and expressed as the p27^Kip1^/DAPI ratio. The data indicated a 140% p27^Kip1^ increase at 3 days (Figure 3A, adjusted *p* = 0.0045), a 128% value at 5 days (Figure 3B, adjusted *p* = 0.0399) and a 122% value at 7 days (Figure 3C, adjusted *p* = 0.0471). In all cases, the results indicated statistically significant data.

**FIGURE 4:**
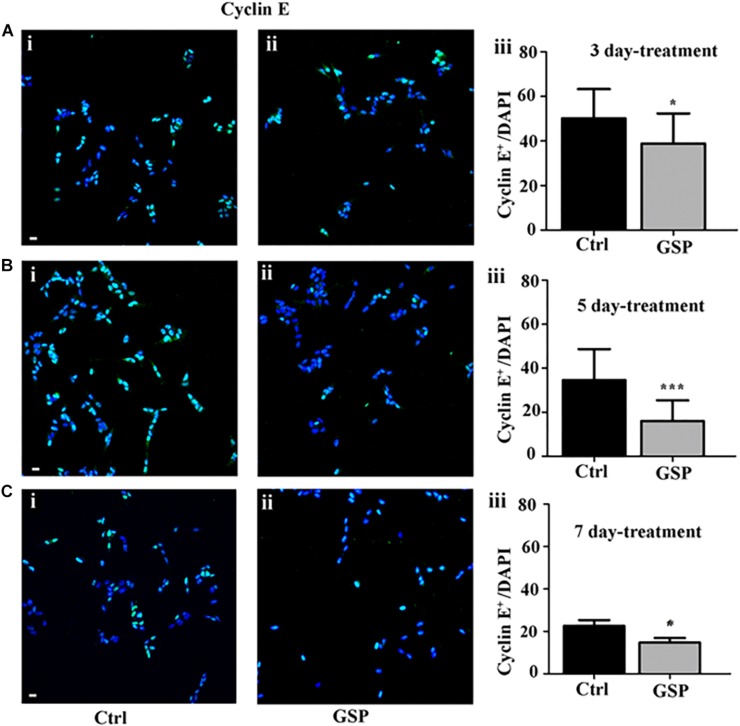
GSP treatment counteracts Cyclin E expression in SH-SY5Y neuroblastoma cells. SH-SY5Y Neuroblastoma cell proliferation was evaluated through expression of cyclin E, reported in the absence (Ctrl, **i**) or in the presence of GSP (GSP, **ii**) upon 3 **(A)**, 5 **(B)**, and 7 **(C)** days of treatment. In panels **(A–C,iii)**, the bar graphs compare the Cyclin E/DAPI ratio in the above conditions. The data are representative of at least three experiments, and values are expressed as the mean ± SEM. Statistical analysis was done through a *t*-test: ^*^p < 0.05; ^∗∗∗^*p* < 0.0001. Scale bars: 20 μm.

**FIGURE 5:**
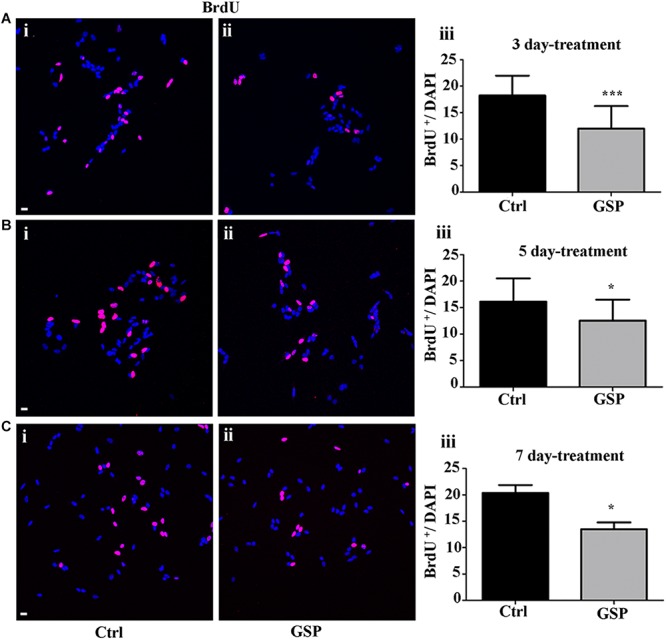
GSP treatment counteracts BrdU incorporation in SH-SY5Y neuroblastoma cells. BrdU incorporation is reported in GSP-treated (GSP, **ii**) versus control (Ctrl, **i**) SH-SY5Y cells upon 3 **(A)**, 5 **(B)**, and 7 **(C)** days of treatment. Bar graphs **(A–C,iii)** compare the number of double BrdU/DAPI labeled cells in both conditions upon 3 **(Aiii)**, 5 **(Biii)**, and 7 **(Ciii)** days of treatment. Data are representative of at least three experiments and the values report the mean ± SEM. For statistical analysis a *t*-test was used: ^*^p < 0.05; ^∗∗∗^*p* < 0.0001. Scale bars: 20 μm.

The polyphenol effects on cell cycle progression were also evaluated during the G1 phase of the cell cycle through the expression of Cyclin E, a marker of the G1/S transition (Figure 4) and by BrdU incorporation, which labels cells in the S phase (Figure 5). Expression of the above markers was again evaluated through the number of labeled versus the overall cell number (estimated by DAPI staining) before comparing the different conditions. At 3 days, Cyclin E expressing cells were 77% in treated versus control samples (Figure 4A, adjusted *p* = 0.0273), which at 5 days become 46% (Figure 4B, adjusted *p* = 0.0003), and at 7 days 65% (Figure 4C, adjusted *p* = 0.0286).

In Figure 5, the extent of BrdU labeling on proliferating cells is shown at the different incubation times (3, 5, and 7 days). The percentage BrdU labeled cells in GSP-treated versus control cells was 66% in 3 day cultures (Figure 5A, adjusted *p* = 0.0003) while at 5 days a higher percentage (77.6%) was observed (Figure 5B, adjusted *p* = 0.018) and a 66.0% value was again observed at 7 days (Figure 5C, adjusted *p* = 0.047). In all cases Bonferroni corrected *p*-values were used.

Statistically significant decreases of both markers were therefore demonstrated in GSP-treated versus control cells, thus indicating inhibitory effects of GSP on cell cycle transition (Figures 4Aiii,Biii,Ciii, 5Aiii,Biii,Ciii).

##### Analysis of the neuronal phenotype

**FIGURE 6:**
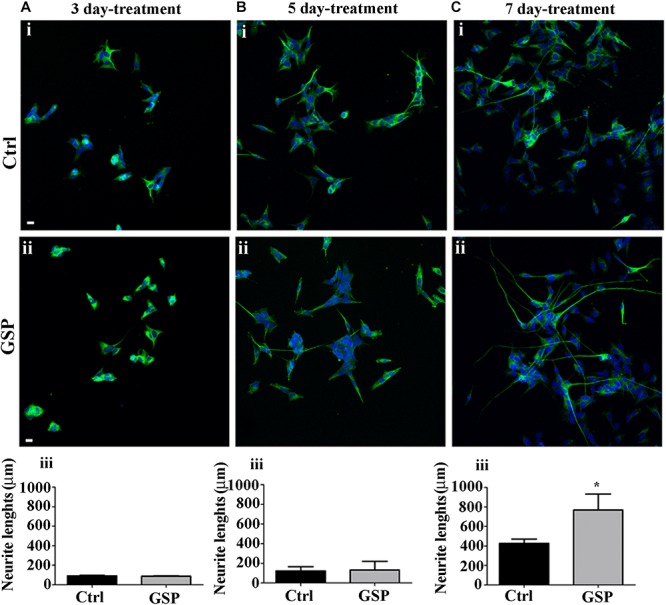
Neuroblastoma differentiation is promoted by GSP treatment. Neuroblastoma cell differentiation along the neuronal lineage is demonstrated through expression of the NeuN neuronal marker in GSP-treated **(ii)** versus control **(i)** SH-SY5Y cells upon 3 **(A)**, 5 **(B)**, and 7 **(C)** days of treatment. Neuronal differentiation was deduced by measuring neurite lengths in SH-SY5Y cells in GSP-treated **(ii)** versus control **(i)** cells. In panels **(A–C,iii)**, bar graphs compare neurite length in cells treated with GSP versus control cells for 3 **(Aiii)**, 5 **(Biii)**, and 7 **(Ciii)** days. Data are representative of at least three experiments, and values are expressed as the mean ± SEM. For statistical analysis a *t*-test was used: ^*^p < 0.05. Scale bars: 20 μm.

The effects on Cyclin E expression and on BrdU incorporation were both congruent with the observed changes in p27^Kip1^ expression as they indicated increased cell cycle exit and reduced proliferation of GSP-treated cells. These data, then, predicted positive effects on neuronal precursor commitment. To confirm this possibility, differentiation along the neuronal lineage was estimated through the expression of β-tubulin and by morphometrically measuring neurite length through the NeuriteTracer, an ImageJ plug-in: the arising data were then statistically evaluated through the Student’s *t*-test. As shown in Figure 6, GSP treatment resulted in a faint, although detectable increase in neurite length in treated versus control cells already in 3 and in 5 days old cultures (Figures 6A,B). On the other hand, sharper effects were observed at 7 days, when a 180% (adjust *p* = 0.03) increase in neurite elongation confirmed the positive effects of GSP treatment along neuronal lineage differentiation (Figure 6C). The above data clearly indicated a relevant increase in the differentiation/proliferation ratio of SH-SY5Y cells as a consequence of GSP treatment. Based on the above findings it could then be inferred that in a cell line of neuronal derivation, GSP treatment promoted the differentiated phenotype while counteracting proliferation.

To confirm this trend an available *in vivo* model of developmental delay was chosen, which consisted in the mentioned TAG/F3 transgenic line (Bizzoca et al., 2003), in which the effects of GSP treatment on nervous tissue (cerebellar) development were explored.

#### *In vivo* Approach: Polyphenol Effects on Cerebellar Development of Contactin1-Overexpressing Transgenic Versus Control Mice

**FIGURE 7:**
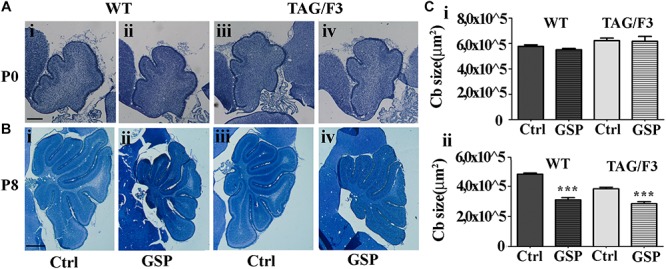
GSP treatment affects cerebellar size in newborn (P0) and postnatal day 8 (P8) mice. **(A,B)** Toluidine blue-stained sagittal sections from either WT **(i,ii)** and TAG/F3 transgenic **(iii,iv)** mice cerebellum in the absence **(i,iii)** or in the presence **(ii,iv)** of GSP treatment. In panel **(C)**, the result of the morphometric analysis of the stained sections surfaces is reported. The data are the result of the analysis of 40 cerebellar sections from 16 mice arising from either the WT and the TAG/F3 genotypes (8 wt and 8 TAG/F3). **(A)** Low magnification images (5×) arising from WT **(i,ii)** and TAG/F3 **(iii,iv)** newborn mice. Scale bar: 200 μm. **(B)** Low magnification images (2.5×) arising from WT **(i,ii)** and TAG/F3 **(iii,iv)** mice at P8. Scale bar: 500 μm. **(C)** Bar graphs comparing the surface of the cerebellar sections at P0 **(i)** and P8 **(ii)** from either WT and TAG/F3 genotypes in either control (Ctrl) or GSP-treated (GSP) mice, through a two-wayANOVA test. Asterisks indicate statistically significant differences: ^∗∗∗^*p* < 0.0001.

To explore the polyphenol effects on cerebellar development, the phenotypes of TAG/F3 and WT mice in the presence and in the absence of GSP were compared at P0 and P8, when the overall cerebellar size, as well as the expression of proliferation/differentiation markers were morphometrically estimated. As for the cerebellar size, in newborn mice no significant differences were observed in this parameter in either genotype in polyphenol-treated versus control mice (Figure 7A, compare i with ii and iii with iv and Figure 7Ci). At postnatal day 8, previous studies performed over a developmental window spanning the first postnatal week revealed a developmental delay in TAG/F3 versus WT mice (Bizzoca et al., 2003), which could be deduced from the reduced surface of cerebellar sections. In the present study, such an effect was confirmed, with a consistent reduction of the cerebellar size at P8 in TAG/F3 mice (Figure 7B, compare i with iii and Figure 7Cii). At this developmental stage, GSP treatment led to an additional reduction in both genotypes (Figure 7B, compare i with ii and iii with iv and Figure 7Cii). The main GSP effects were observed on the WT genotype, while minor although still significant differences could be detected in the TAG/F3 littermates. Indeed, in GSP-treated WT mice a cerebellar size of 64% compared to untreated controls (adjusted *p* < 0.0001) was demonstrated while in TAG/F3 littermates this value accounted to 74% (adjusted *p* < 0.0001). While reflecting reduced cerebellar size in TAG/F3 mice compared to controls (Bizzoca et al., 2003), these data indicated that at this stage polyphenol treatment exerted lower effects in transgenic mice compared to WT littermates. Overall, these data indicated that Contactin1 overexpression and polyphenol administration both negatively modulated cerebellar size with stronger effects of polyphenols compared to those of Contactin1. This suggested that these effects were exerted through the same pathways and given the Contactin1 ability to activate the Notch pathway (Hu et al., 2003, 2006; Bizzoca et al., 2012), that GSP and Contactin1 could exert their effects through such a pathway.

To further explore the mechanisms potentially responsible for the reported GSP effects on the cerebellar size, neuronal precursor proliferation and commitment/differentiation were separately evaluated. In the TAG/F3 line, NeuN expression (to estimate Neuronal commitment) and the density of double NeuN/BrdU positive elements (for neuronal commitment of proliferating precursor) were estimated and compared to control mice.

**FIGURE 8:**
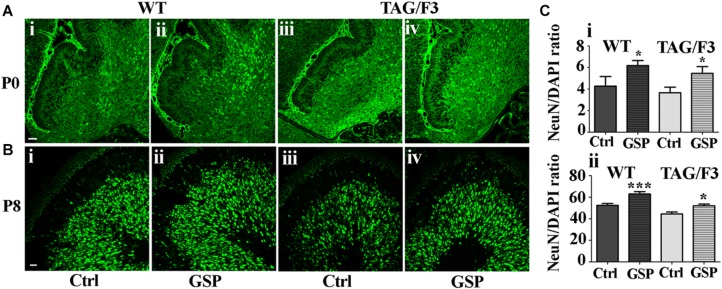
Cerebellar neurogenesis is promoted in WT **(A,B,i,ii)** and in TAG/F3 **(A,B,iii,iv)** transgenic mice upon GSP treatment, as demonstrated by NeuN immunostaining. Cerebellar sections from either untreated **(i,iii)**, or GSP-treated **(ii,iv)** mice were analyzed upon NeuN immunostaining at both P0 (**A**, 20× magnification, scale bar 40 μm) and P8 (**B**, 40× magnification, scale bar 20 μm). **(C)** Bar graphs showing the results of the morphometric analysis of the immunostaining shown in panels **(A,B)**, reporting the number of NeuN/DAPI positive cells ratio at P0 **(i)** and P8 **(ii)**. Asterisks indicate statistically significant differences: ^*^p < 0.05; ^∗∗∗^*p* < 0.0001 through a two-way ANOVA test.

As shown in Figure 8, neuronal commitment, was significantly increased upon polyphenol treatment in both newborn WT (144% adjusted *p* = 0.046) and TAG/F3 (149% adjusted *p* = 0.032) mice (Figure 8A, compare i with ii and iii with iv, and Figure 8Ci). Such a profile was maintained at postnatal day 8, although lower effects of GSP treatment were observed in both WT (120% adjusted *p* = 0.0004) and TAG/F3 (117%, adjusted *p* = 0.0205) mice (Figure 8B, compare i with ii and iii with iv and Figure 8Cii).

**FIGURE 9:**
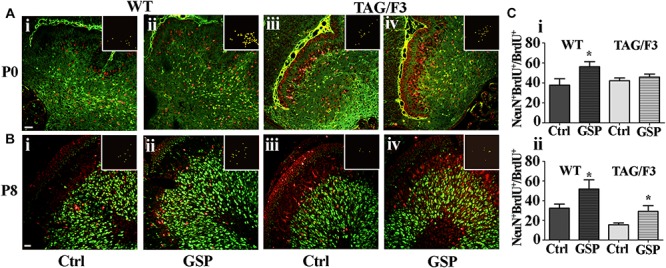
Cerebellar precursors neuronal commitment is promoted in newborn and postnatal day 8 WT **(A,B,i,ii)** as well as in P8 **(B,iii,iv)**, but not in newborn **(A,iii,iv)** TAG/F3 mice in GSP treated **(ii,iv)** versus untreated **(i,iii)** controls (Ctrl). The data are deduced in sagittal sections from both genotypes and morphometrically estimated as the ratio of double NeuN-BrdU versus BrdU immunostaining. **(A)** 20× magnification images of P0 cerebellar cortex, scale bar: 40 μm. **(B)** 40× magnification images of P8 cerebellar cortex, scale bar: 20 μm. **(C)** Bar graphs showing the results of the morphometric analysis of the immunostaining shown in panels **(A,B)**, reporting the ratio among the number of double NeuN-BrdU versus BrdU-labeled cells at the selected developmental stages: P0 **(i)** and P8 **(ii)**. Colocalization points are reported in the insets as ImageJ plug-in output. In panel **(C)**, asterisks indicate statistically significant differences: ^*^p < 0.05 through a two-way ANOVA test.

In both WT and TAG/F3 mice the polyphenol effects were also morphometrically estimated on precursor commitment toward the neuronal lineage upon double NeuN-BrdU immunostaining at both P0 and P8 and expressed as the NeuN-BrdU versus BrdU ratio. In newborn WT cerebellum, GSP treatment resulted in positive effects on proliferating precursor commitment toward the neuronal lineage (148%, adjusted *p* = 0.0309; Figures 9Ai,ii,Ci) while very minor, non-significant effects were observed in newborn TAG/F3 mice (Figures 9Aiii,iv,Ci). In postnatal day 8 cerebellum, precursor commitment toward the neuronal lineage was still sharply increased in WT mice (160%, adjusted *p* = 0.036) upon polyphenol treatment (Figures 9Bi,ii,Cii) with sharper effects in TAG/F3 littermates (188%, adjusted *p* = 0.037; Figures 9Biii,iv,Cii).

These data indicated a general positive effect of GSP treatment on neuronal precursor commitment at both P0 and P8 in WT mice. In TAG/F3 littermates very low non-significant GSP effects were observed in newborn animals while a sharp increase was demonstrated at P8, confirming the generally stimulatory effects of polyphenols on neurogenesis at this stage and in this genotype. Therefore, overall, the data indicated differential polyphenol effects on neurogenesis depending upon the developmental stage and the genetic background, the effects being mostly demonstrated in Contactin 1-overexpressing mice at the end of the first postnatal week. In turn this indicated that at this stage Contactin1 overexpression could promote nervous tissue responsiveness toward polyphenols.

**FIGURE 10:**
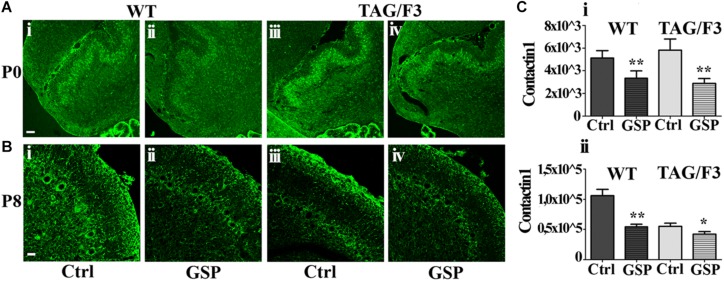
Contactin1 expression is counteracted at P0 and P8 in sagittal cerebellar sections from the developing WT **(A,B,i,ii)** and TAG-F3 **(A,B,iii,iv)** mice in the presence (GSP, **ii,iv**) versus the absence (Ctrl, **i,iii**) of GSP treatment. **(A)** 20× magnification images of P0 cerebellar cortex **(i–iv)**. Scale bar: 40 μm. **(B)** 40× magnification images of P8 cerebellar cortex **(i–iv)**. Scale bars: 20 μm. **(C)** Bar graphs showing the results of the morphometric analysis of the immunostaining shown in panels **(A,B)**, reporting the Contactin1 positive pixel per field at the selected developmental stages: P0 **(i)** and P8 **(ii)**. Asterisks indicate statistically significant differences: ^*^p < 0.05; ^∗∗^*p* < 0.001 through a two-way ANOVA test.

We also wanted to evaluate whether GSP treatment affected Contactin family members (both Contactin1 and Contactin2) expression in the different genotypes. As for Contactin1, at P0 its expression was significantly downregulated in WT (to a 65% value, adjusted *p* = 0.006) and mostly in TAG/F3 (to a 49% value, adjusted *p* = 0.0024) mice upon GSP treatment (Figures 10A,Ci). This indicated general inhibitory effects of such polyphenol on Contactin1 expression, with the strongest effects in TAG/F3 mice, likely reflecting the higher original Contactin1 levels in this line.

At P8 strong GSP effects were still observed on WT mice (51%, adjusted *p* = 0.0016), while lower, non-significant consequences of the same treatment were observed on TAG/F3 littermates (76%, adjusted *p* = 0.047), likely reflecting the lower endogenous Contactin1 expression at this stage in this genotype as a consequence of developmental delay (see also Bizzoca et al., 2003) (Figures 10Bi,ii,Cii). In TAG/F3 mice, therefore, the differences were much lower, and provided with lower significance in GSP-treated versus control mice (Figures 10Biii,iv,Cii). Overall, these data indicated that GSP treatment resulted in Contactin1 downregulation in newborn mice irrespective of the genotype.

**FIGURE 11:**
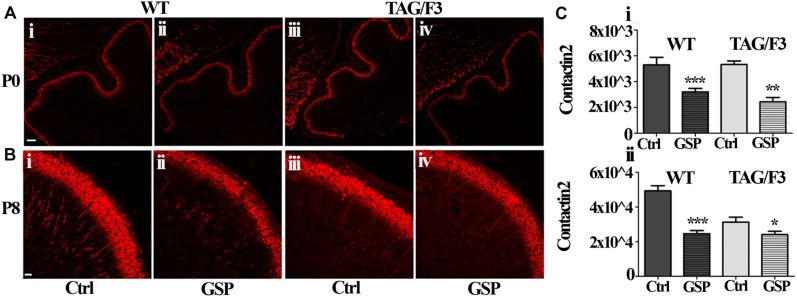
Contactin2 expression is counteracted in sagittal cerebellar sections from developing WT **(A,B,i,ii)** and TAG-F3 **(A,B,iii,iv)** mice in the presence **(ii,iv)** versus the absence **(i,iii)** of GSP. **(A)** 20× magnification of P0 cerebellar cortex. Scale bars: 40 μm. **(B)** 40× magnification of the P8 cerebellar cortex. Scale bars: 20 μm. **(C)** Bar graphs showing the results of the morphometric analysis of the immunostaining shown in panels **(A,B)**, reporting the area of Contactin2 positive pixel per field at P0 **(i)** and P8 **(ii)**. Asterisks indicate statistically significant differences: ^*^p < 0.05; ^∗∗^*p* < 0.01; ^∗∗∗^*p* < 0.001 through a two-way ANOVA test.

As shown in Figure 11, similarly to Contactin1, a sharp decrease in Contactin2 expression was observed in newborn cerebellum upon GSP treatment both in WT (60%; adjusted *p* < 0.0001) and TAG/F3 (45%; adjusted *p* = 0.0025) mice (Figures 11A,Ci). At postnatal day 8, a sharp decrease in Contactin2 expression was similarly demonstrated upon GSP treatment in WT mice cerebellum (49%; adjusted *p* < 0.0001) while lower effects were observed in TAG/F3 littermates (78%; adjusted *p* = 0.041) (Figures 11B,Cii).

Therefore, although at different extents, the expression of Contactin1 and Contactin2 were both downregulated as a consequence of GSP treatment in WT and TAG/F3 mice. Sharper effects were found to occur for both Contactins at P0 while lower consequences were observed for Contactin2 at P8 in TAG/F3 mice, likely reflecting its lower expression levels at this stage in this genotype.

##### Relationships between contactins expression and notch pathway activation

**FIGURE 12:**
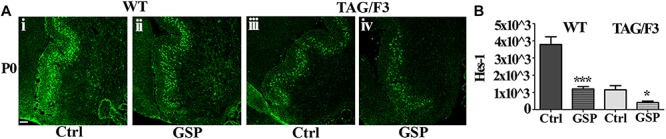
Expression of the Hes-1 transcription factor is counteracted by polyphenol (GSP) treatment in cerebellar sections from WT and TAG/F3 mice cerebellum. **(A)** Hes-1 transcription factor expression in sagittal sections from the developing cerebellum from WT **(i,ii)** and TAG/F3 **(iii,iv)** mice upon GSP treatment **(ii,iv)** versus control **(i,iii)** conditions, shown in 20× magnification images. Scale bar: 40 μm. **(B)** Bar graphs showing the results of the morphometric analysis of the immunostaining shown in panel **(A)**, reporting the Hes-1 positive pixel per field in control (Ctrl) conditions and upon GSP treatment (GSP) in WT and in TAG/F3 mice. Asterisks indicate statistically significant differences: ^*^p < 0.05; ^∗∗∗^*p* < 0.0001, through a two-way ANOVA test.

As anticipated, besides by its canonical ligands, the Notch pathway may be also activated by adhesion molecules of the IgSF and in particular by components of its Contactin subset, typically shown in the case of Contactin1 (Hu et al., 2003; Bizzoca et al., 2012). Therefore, in the present study, Notch pathway activation was estimated through the Hes-1 transcription factor expression profile and compared in WT and TAG/F3 mice upon GSP administration (Figure 12A). Morphometric analysis revealed that, irrespective of the genotype, GSP treatment resulted in a sharp Hes-1 and therefore Notch pathway downregulation in both genotypes, with in particular a 32% in WT GSP treated (adjusted *p* < 0.0001) compared to control mice, while this percentage was raised to 35% in TAG/3 littermates (adjusted *p* = 0.021) (Figure 12B). In addition, comparing the consequences of the GSP administration and of Contactin1 expression revealed that the two conditions resulted in similar effects (Figure 12B). Finally, such effects were found to be cumulative, strongest effects being observed in TAG/F3 mice upon GSP treatment. Given that Contactin1 effects on neurogenesis are known to imply Notch pathway activation, a likely explanation could be that GSP treatment exerted its effects by counteracting Contactin1 expression and as such Notch pathway activation.

## Discussion

In this study the mechanisms underlying neuronal developmental parameters and their modulation by adhesion molecule expression and by polyphenol administration are explored through the use of both *in vitro* and *in vivo* approaches. As for the former, in the SH-SY5Y cell line the consequences are explored of polyphenol treatment on both precursor proliferation and on their neuronal commitment/differentiation. A related aspect concerns the significance in such events of axonal adhesive glycoprotein expression profile, for which an *in vivo* approach was rather devised, which took advantage of using transgenic mice models of adhesive glycoproteins mis- and over-expression, known to result into a developmental delay (Bizzoca et al., 2003, 2012), which represented a valuable tool for testing protective treatments.

### Regulatory Pathways of Neural Developmental Control

The central outcome of this study concerns the control pathways involved in the role of both adhesive glycoproteins and polyphenols in developing nervous tissue patterning, the Notch pathway representing a relevant link between them (Louvi and Artavanis-Tsakonas, 2006; Pierfelice et al., 2011; Wang, 2011; Dhanesh et al., 2016). Indeed, neural development implies the coordination among a wide set of processes during both early and late neurogenesis (Ishibashi, 2004; Barros et al., 2011; Paridaen and Huttner, 2014; Hardwick et al., 2015; Clément et al., 2018), which depends upon precursor interactions with either the surfaces of flanking cells and/or extracellular matrix components (Schmidt and Rathjen, 2010; Hirano and Takeichi, 2012; Frei and Stoeckli, 2014; Petrovic and Schmucker, 2015). Such interactions are critical for tissue morphogenesis as they modulate precursor differentiation (Pfeuty, 2015; Kalcheim, 2018) through activation of signaling pathways (Kanemoto et al., 2014), among which a key role is played by the one associated with Notch receptors (Ruijtenberg and van den Heuvel, 2016). Activation of Notch receptors typically results from their interaction with specific ligands, originally identified in the canonical Delta and Serrate proteins (Stump et al., 2002; Steinbuck and Winandy, 2018). In turn, such interactions activate a γ-secretase-dependent proteolytic processing, responsible for generating the Notch intracellular domain (NICD), which, upon nuclear translocation, then activates developmental control pathways (Bergmans and De Strooper, 2010). As a general rule, proteolytic processing may also be relevant in modulating adhesive glycoproteins activation and biological function as typically postulated in the case of at least two components of cell adhesion molecules families: the L1 glycoprotein (Kiefel et al., 2011; Kataria et al., 2016) as well as Cadherin family components (Paradies and Grunwald, 1993; Murata et al., 2014; Batchuluun et al., 2017), supporting the hypothesis that besides the canonical Notch pathway also non-canonical Notch activation by structurally different proteins may be involved in its function (D’Souza et al., 2010).

Among the non-canonical activators, a subset of the immunoglobulin (Ig) supergene family has to be also mentioned, which includes molecules built by the association of Immunoglobulin type C2 with Fibronectin type III (FNIII) repeats (IgC2/FNIII molecules). Such an association is of structural and functional relevance given the similar overall organization of such domains (Cota et al., 2001); in addition, a peculiarity of such molecules concerns their mode of membrane association, which implies C-terminal GPI-containing lipid anchors, allowing their high level mobility therein and, in addition, their delivery in soluble form. Such an overall organization is demonstrated for all the six Contactin family components, labeled from Contactin1 to Contactin6, in turn characterized by differential expression profiles and therefore developmental roles (Zuko et al., 2011).

This study focuses on two components of this subset, Contactin1 (Ranscht, 1988; Gennarini et al., 1989; Bizzoca et al., 2003, 2012; Hu et al., 2003) and Contactin2 (Furley et al., 1990), which display a high level of structural similarity, resulting in a nearly 50% homology in the aminoacid sequences. However, the genes encoding these two molecules sharply differ from each other in terms of their activation profiles, as *Cntn2* peaks at the time of precursor cell cycle exit and neuronal commitment while *Cntn1* is mostly activated in later developmental stages on growing axonal extensions. These data strongly support the hypothesis that the functional and developmental specificity of such molecules largely depends upon the differential activation profiles of the underlying genes.

### Contactins Regulatory Functions

Based on the complexity of the mechanisms which drive their regulated expression it has been proposed that Contactins, originally classified among the cell adhesion molecules, should rather fulfil the more general definition of morphoregulatory molecules (Edelman and Jones, 1997; Gennarini et al., 2017) as, rather than cell adhesion *per se*, their role implies transmembrane signaling pathways activation. The Notch pathway is part of this morphoregulatory role mostly during the early development when its activation finely modulates the balance of neuronal precursor proliferation versus differentiation events (Zhang et al., 2018).

### Contactin Role in Developmental Control: Molecular and Cellular Bases

In such a mechanism cell cycle control plays a major role. Indeed, at the cellular level, the Notch pathway primary function mostly concerns S phase modulation through the control of cell cycle entry/exit balance and therefore its primary developmental function is to modulate proliferation of precursor cells by shortening their persistence into the G1 phase, thus promoting S phase entry. These effects imply transcriptional activation of the S phase kinase-associated protein SKP2, a subunit of the ubiquitin-ligase SCF^SKP2^ complex whose upregulation enhances proteasome-mediated degradation of the Cyclin Kinase inhibitors (CKIs) p21^Cip1^ and p27^Kip1^ (Bornstein et al., 2003; Sarmento et al., 2005), which in turn promotes precursor cell cycle entry and therefore proliferation. At the same time, precursor migration is also promoted, which contributes to the overall positive effects on neurogenesis (Clément et al., 2018) by modulating the balance of proliferation versus differentiation events (Ishibashi, 2004; Nelson et al., 2007). Given that the Notch pathway is activated by interactions with Contactin1 (Hu et al., 2003, 2006; Bizzoca et al., 2012), it may then be inferred that a key developmental function of Contactin proteins and of the associated Notch pathway is to mediate the control of tissue patterning, including precursor cell cycle exit, migration and differentiation (Imayoshi et al., 2013; Dhanesh et al., 2016; Zhang et al., 2018).

### Significance of Contactin/Notch Interactions in Modulating the Nutritional Input

The above evidences support the known Contactin1 regulatory role in neural developmental control. However, a further relevant aspect arising from the present study concerns the consequences of Contactin/Notch interactions in modulating the effects of the nutritional input, which may still depend upon the activation of specific signaling pathway. It is well known that the mother’s diet influences fetal and in particular neural development through components of the intestinal microbiota which, in turn, may prevent disease outcome and, in particular, neurological disorders, even including autism (Macpherson et al., 2017; Ficara et al., 2018; Roman et al., 2018). Therefore, a relevant aspect of the present study concerns the role of Notch receptors and of the associated signaling pathway in modulating the effects of the nutritional input (Mori et al., 2017, 2018). Indeed, polyphenols interactions with Notch receptors are known to counteract their function and therefore γ-secretase activation, which results in positive effects on neurogenesis, thus promoting the differentiated phenotype and preventing neuronal damage (Gu et al., 2015). Inhibition of Notch pathway through the specific effects of polyphenols on γ-secretase therefore may account for the positive effects of such compounds on neurogenesis, which in turn justifies their protective effects against neurodevelopmental delay and neurodegeneration (Donato et al., 2014).

On this basis, a relevant outcome of the present study is devoted to understanding the mechanisms whereby polyphenols modulate the Notch pathway at the tissue level, which has been addressed through the use of both *in vitro* and *in vivo* approaches.

### *In vitro* Approaches

In the SH-SY5Y cell line the effects of polyphenol administration on the cell cycle have been explored (Shin et al., 2013). Polyphenol-treated SH-SY5Y cells underwent upregulation of the CIP Kip family member p27^Kip1^, known to drive precursor cell cycle exit toward the G0 phase at the G1/S interphase (Iacovelli et al., 2007). These effects are consistent with those exerted on further cell cycle regulators/markers, as indicated by the decrease of Cyclin E expression and of BrdU incorporation in precursor cells. These effects begun at the third day of SH-SY5Y cells polyphenol treatment, a time lapse necessary for activating expression and setting up of molecules mediating cell cycle control and they justify the observed increase in cell cycle exit, demonstrated through expression of neuronal commitment markers and, morphologically, by increased neurite elongation. As for the underlying mechanisms, besides promoting cell cycle exit, p27^Kip1^ may also drive neuronal precursor differentiation through its cyclin kinase inhibitory functions (Tury et al., 2011, 2012) and, in addition, through its ability to suppress RhoA signaling, based on the property of the latter in modulating the neuronal cytoskeleton, thus promoting neuronal differentiation (Clément et al., 2018).

### *In vivo* Approaches

In the present study, further data arose from the use of an *in vivo* model consisting in transgenic lines undergoing developmental delay as a consequence of Contactin1 over-expression (Bizzoca et al., 2003, 2012, 2015), in which the consequences were studied of dietary polyphenol administration. The effects of GSP used as a dietary polyphenol source were explored in modulating neurogenesis by using as a model TAG/F3 mice, undergoing Contactin1 over-expression and Notch pathway upregulation, in turn resulting into a neurodevelopmental delay (Bizzoca et al., 2003, 2012). In this transgenic line specific effects including increased precursor proliferation and reduced neuronal commitment/differentiation in different neural regions, including the cerebellum (Bizzoca et al., 2003), the cerebral cortex (Bizzoca et al., 2012), the basal ganglia (Massaro et al., 2012) and the hippocampus (Puzzo et al., 2013) were observed. Therefore it represented a valuable model of impaired neural development, which could mimic the consequences of neurodegenerative disorders, and in which the effects could be tested of polyphenol treatment. Indeed, in this line the inhibitory Contactin1 effects on neurogenesis were efficiently counteracted by polyphenol treatment (see for instance Figures 8, 9) and the involvement in this phenotype of the Notch pathway was demonstrated through the concomitant decrease in Hes-1 expression (Figure 12). These effects persisted throughout development as they could be demonstrated at both P0 and P8. However, they were mostly observed at the end of the first postnatal week, when reduced cerebellar size correlated to Contactin1 overexpression. At both developmental stages, an increase in neuronal commitment resulted from polyphenol treatment, mostly evident in Contactin1-overexpressing mice at postnatal day 8. Therefore, these polyphenol-dependent effects in TAG/F3 mice were consistent with those exerted on the Notch pathway.

### Polyphenol Effects on Contactins and Hes-1 Expression

Consistent relevant data arising from the present study concern the polyphenol-dependent decrease of both Contactin1 and Contactin2 expression arising from polyphenol treatment in both genotypes and in both considered developmental stages (P0 and P8). Since Contactin1 is an alternative ligand of Notch (Hu et al., 2003, 2006; Bizzoca et al., 2012), a decrease in its expression could account for the observed decrease in Notch activation, deduced from the concomitant decrease in Hes-1 expression. However, such polyphenol effects do not seem to be specific for Contactin1 as a Contactin2 decrease was observed at the same time in TAG/F3 mice. The inhibitory polyphenol effect on adhesion molecules expression is known in the literature, and it arises from studies concerning the relationships between redox cell state and adhesion molecules expression (Carluccio et al., 2003). It is worth mentioning that the observed effects on Contactins expression were consistent with those observed on the cerebellar size, also reduced at P8 (see Figure 7), thus supporting the hypothesis that the primary effects of polyphenol treatment was on the adhesion protein expression itself and on the subsequent precursor commitment toward the neuronal phenotype.

Based on the above findings, it may then be interesting to interpret the polyphenol effects on neuronal precursors changes observed on the cerebellar neurons in relation to those on the Notch pathway. In the TAG/F3 model, previous studies demonstrated that Contactin1 overexpression correlates with a Notch pathway activation, with a concomitant decreased expression of cell cycle exit (p27^Kip1^) and neuronal commitment and increased expression of proliferation (BrdU) markers (Bizzoca et al., 2012), thus justifying positive Contactin1 effects on precursor proliferation and negative effects on cell cycle exit and neuronal commitment, which resulted into a developmental delay.

In the present study, the effects of polyphenol administration were similarly explored on Contactins adhesion molecules expression itself, which was clearly reduced as a consequence of polyphenol treatment. Given the positive Contactin1 effects on the Notch pathway, this resulted in opposite effects on neuronal commitment, so that, in the presence of polyphenols, cell cycle exit was clearly promoted as demonstrated by the increase in both p27^*kip*^ and NeuN expression (Figures 8, 9).

These effects could be deduced by the use of both *in vitro* and *in vivo* approaches. In the former, using the SH-SY5Y cells demonstrated positive effects of polyphenol administration on the differentiated phenotype, indicated by increased cell cycle exit and neuronal commitment markers. *In vivo*, in the previously generated model of neural developmental delay represented by the TAG/F3 mice (Bizzoca et al., 2003), phenotype analysis revealed an overall positive effect of polyphenol administration, essentially at postnatal day 8, when the significant decrease in cerebellar neurogenesis demonstrated in TAG/F3 mice, dependent upon Notch pathway activation by Contactin1, was efficiently counteracted upon polyphenol administration.

The link between Contactins expression, Notch pathway activation and Cip/Kip family factors expression is consistent with the literature data (Sarmento et al., 2005), indicating a reverse relationship between the Notch pathway activation and p27^Kip1^ factor expression, which correlates with the inhibitory effects of polyphenols on γ-secretase activity (Gu et al., 2015). It could then be supposed that such a relationship may definitely account for the positive effects of polyphenols on neurogenesis so that treatment with such compounds may efficiently counteract neuronal damage.

Besides the demonstrated effects of Notch pathway activation, future studies will be necessary to investigate whether in the chosen model system polyphenols may exert their function also through the expression of neurogenic factors as BDNF, which contributes to neurogenesis, synaptic growth, and neuron survival (Valente et al., 2009). In this context, the peripheral cytokine network aimed at deciphering the immune homeostasis could be also taken into account based on the possibility that peripheral mediators even including release of nitric oxide (Magrone et al., 2007) are transported to the CNS, thus exerting *in situ* their influence also on the Contactin/Notch pathway.

## Ethics Statement

Animals were bred in “Department of Basic Medical Sciences, Neurosciences and Sensory Organs,” University of Bari Aldo Moro, Italy and experimentation conformed the EU directive 2010/63/EU by following the Italian Ministry of Health (law of March 4, 2014, n. 26) upon the Authorization n.982/2016.

## Author Contributions

SP was involved in setting up the animal models used in the present study on which the whole *in vivo* experimental work was done. AB performed the phenotype analysis of such *in vivo* models. PC analyzed the results obtained on cell lines. TM did comparison of the effects observed in the nervous tissue with those potentially expected in the immune system. EJ and GG coordinated the studies performed in parallel on the nervous and the immune systems.

## Conflict of Interest Statement

The authors declare that the research was conducted in the absence of any commercial or financial relationships that could be construed as a potential conflict of interest.
